# Unilateral arm weakness following retroperitoneal lymph node dissection for testicular germ cell tumour

**DOI:** 10.1002/anr3.12312

**Published:** 2024-07-10

**Authors:** S. Gaikwad, B. Trivedi, S. Gholap

**Affiliations:** ^1^ Tata Memorial Centre Mumbai India; ^2^ Dr D. Y. Patil Medical College, Hospital and Research Centre, Dr D. Y. Patil Vidyapeeth Pimpri, Pune India

**Keywords:** brain neoplasms, neurological manifestations, testicular neoplasms

**Figure 1:**
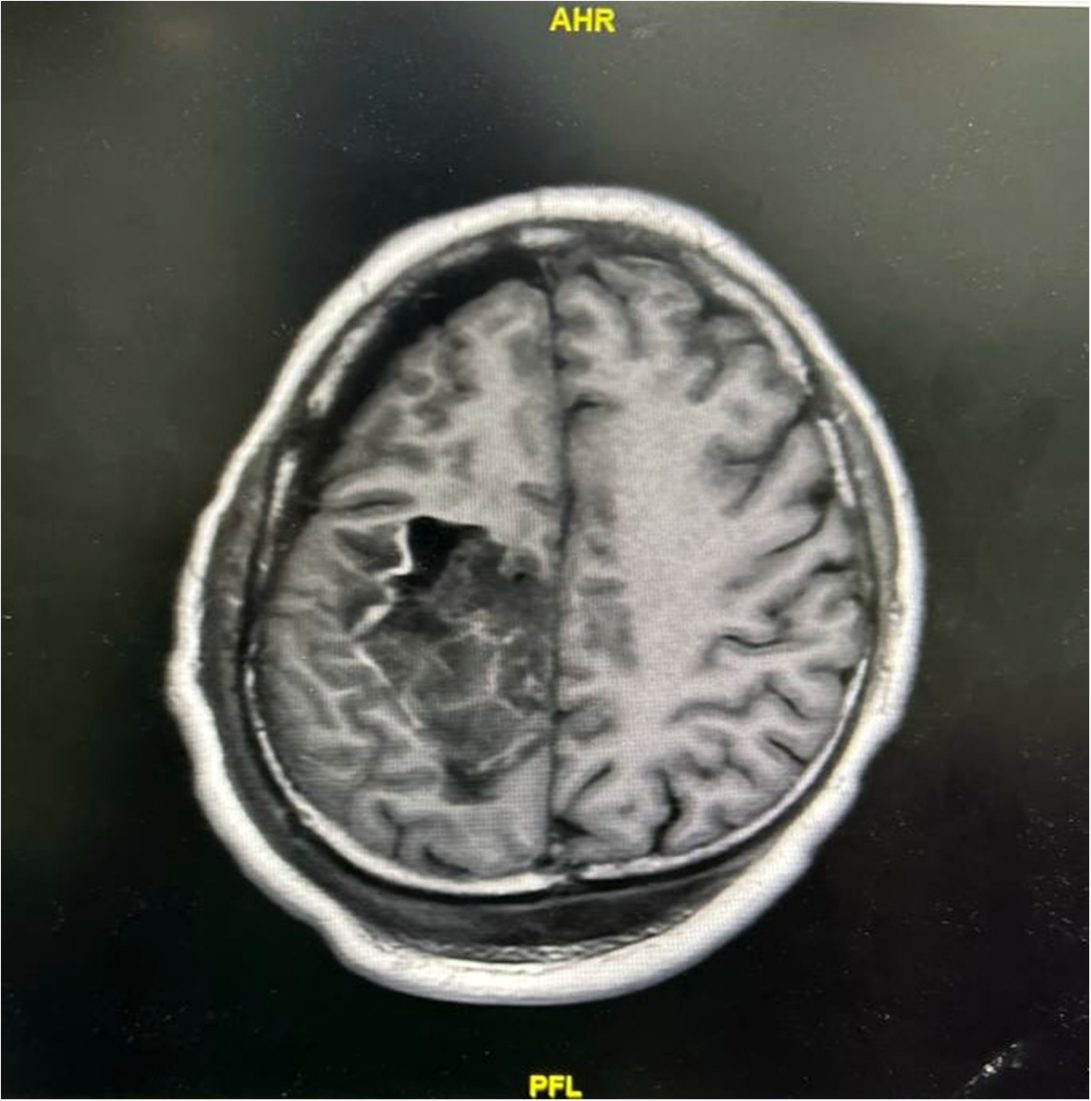
Magnetic resonance imaging of the brain showing a well‐defined, lobulated, enhancing lesion in the right precentral gyrus with mild perilesional oedema.

A 26‐year‐old man underwent retroperitoneal lymph node dissection following diagnosis of non‐seminomatous testicular germ cell tumour. He had previously undergone an inguinal orchidectomy. The surgery was performed under general anaesthesia with thoracic epidural for analgesia. Four hours after the start of surgery, tachycardia and hypotension developed which did not respond to fluid therapy. Therefore, intravenous noradrenaline infusion was started at a rate of 0.01–0.07 μg.kg^−1^.min^−1^ which was discontinued 2 h after surgery. During the operation, both arms were abducted to 90 degrees. At the end of the surgery, the patients' trachea was extubated and he was transferred to the intensive care unit. On the first postoperative day, the patient developed a left upper limb monoparesis without sensory deficit. Magnetic resonance imaging (MRI) of the brain and brachial plexus were undertaken. The MRI brain revealed a haemorrhagic lesion with surrounding oedema, leading to a diagnosis of symptomatic intratumoural bleed with raised intracranial pressure (Fig. 1). This was suspected to be a metastatic lesion as it was well‐defined and solitary. Testicular tumours can metastasise to the brain, although this is rare. The patient was treated with intravenous dexamethasone and received targeted radiotherapy which led to complete recovery of the arm weakness by postoperative day 21.

Brain metastases in non‐seminomatous testicular germ cell tumours are uncommon, but more likely to occur in those over 40, with elevated levels of β‐human chorionic gonadotropin (≥ 5000 IU.l^−1^) and alpha‐fetoprotein (> 10,000 ng.ml^−1^), pulmonary or bone metastases, and neurological symptoms. Due to the absence of these symptoms and low tumour marker levels, pre‐operative brain imaging was not performed in this case [1, 2]. The tumour markers were repeated and were within normal range.

In non‐seminomatous testicular germ cell tumours, spontaneous tumour bleed is rare, and while the patient's coagulation profile and platelets were normal, systemic immune response syndrome and immune suppression may have contributed to the bleeding risk. Systemic immune response syndrome may lead to increased metabolism and vascular complications, possibly influencing the occurrence of intratumoural bleeding [3, 4].

For us, the key point is that brain metastases can mimic anaesthetic complications, such as brachial plexus injury or a cerebrovascular accident. This highlights the need for a comprehensive differential diagnosis in the postoperative period to ensure accurate identification and management of underlying conditions.
